# Supplementary material for: Defense-Related Gene Expression Following an Orthotospovirus Infection Is Influenced by Host Resistance in Arachis hypogaea
Source: Viruses. 2021 Jul 5;13(7):1303. doi: 10.3390/v13071303 (PMC8310252; doi:10.3390/v13071303)
Supplement: Supplementary file 1 [file viruses-13-01303-s001.zip › viruses-1263609-supplementary.pdf]

Supplementary Materials

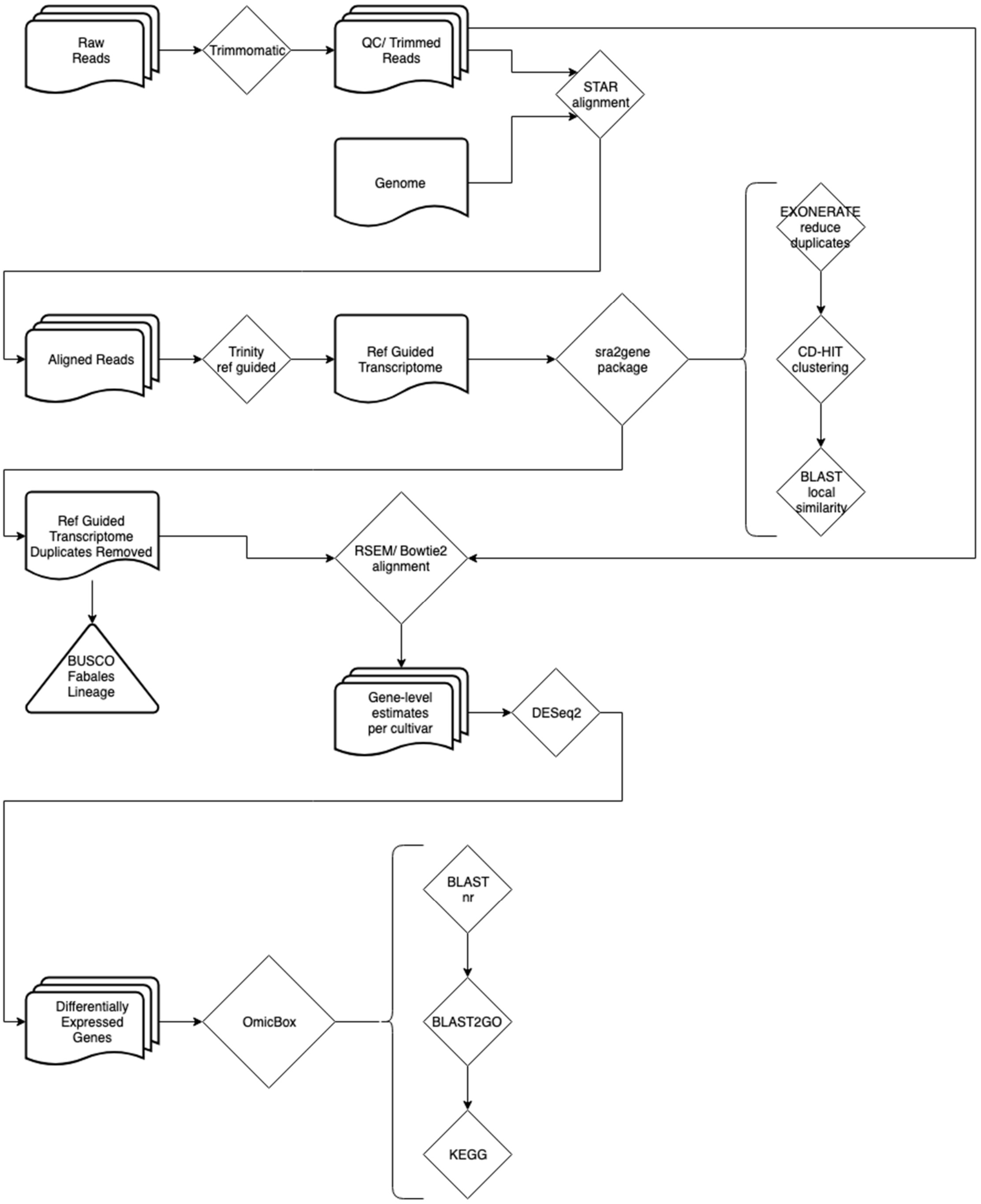

Fig S1. Pipeline for transcriptome assembly. Pipeline for assembly and differential expression analysis. Using the gene models and full allotetraploid genome, this transcriptome assembly incorporated two previously unincluded cultivars. Mapping of raw reads and raw read counts was performed to calculate the levels of expression in non-infected and TSWV-infected peanut plants.

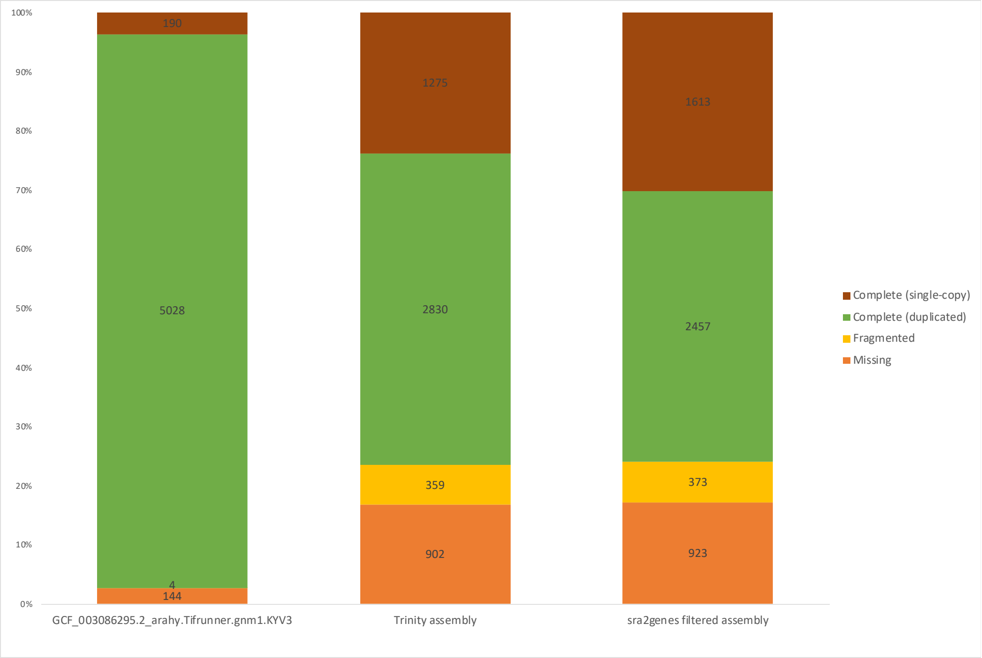

Fig. S2. BUSCO scores from current transcriptome assembly compared to gene models. The total completeness of the transcriptome assembly was checked against the Fabales Odb10 lineage. The total number of complete BUSCOs was 4,070, missing BUSCOs is 923, and fragmented BUSCOs was 373. Of the 4,070 complete BUSCOs, 2,457 were duplicated and 1,613 were single-copy.

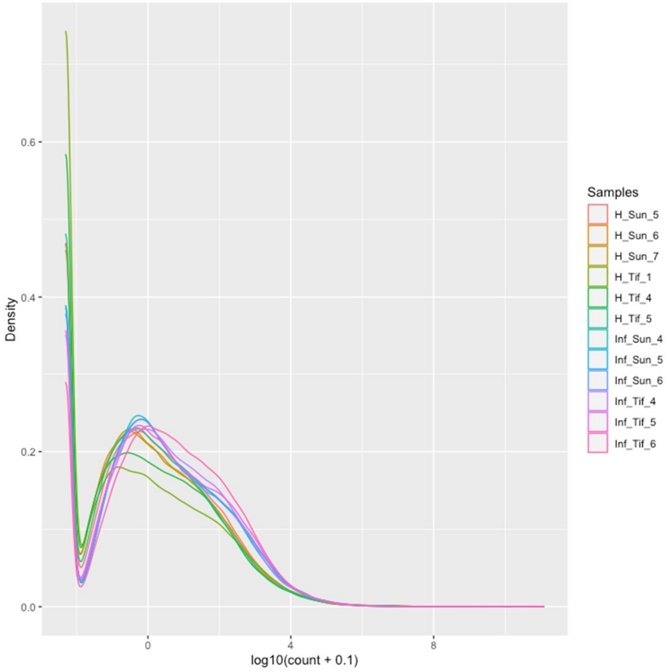

Fig. S3. FPKM across all samples in both cultivars. FPKM of the total number of reads from each sample. Overall sample FPKM was consistent and there were no outliers. Non-infected (H) and infected (I) samples for SunOleic 97R (Sun) and Tifguard (Tif).

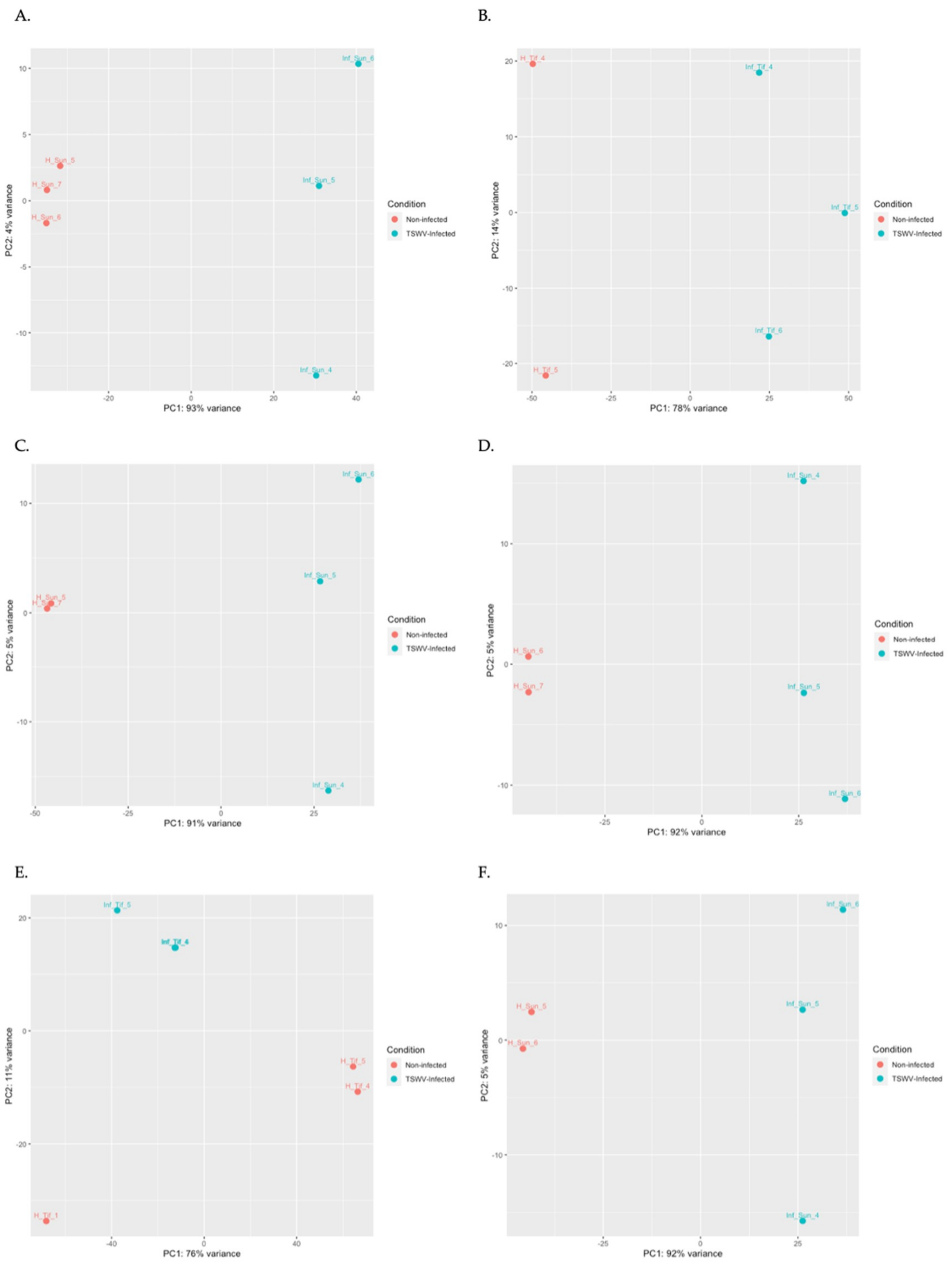

Fig. S4. Comparisons of selective removal of samples to test effects on clustering. Comparisons were conducted to check for stability of sample clustering. (A) SunOleic 97R PCA shows that all samples cluster according to being either healthy or TSWV-infected. (B - D) The removal of one sample from the SunOleic set did not disrupt the clustering of samples. (E) Tifguard PCA with H_Tif_1 included showed poor clustering. (F) The removal of Non-inf_Tif_1 resolves potential confounding effects introduced by the inclusion of H-Tif_1.

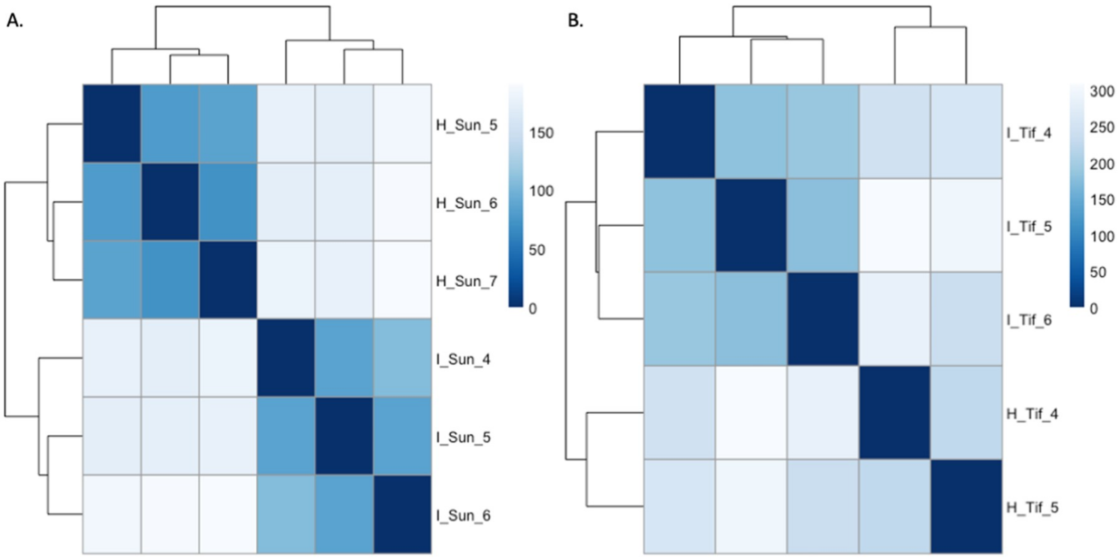

Fig. S5. Sample-to-sample relation within cultivars. Sample-to-sample distances for the SunOleic 97R and Tifguard cultivars, respectively. (A) Samples showed stronger relation to each other based on being either non-infected or TSWV-infected for the SunOleic cultivar. (B) In the Tifguard cultivar, these samples also showed stronger relation to each other based on being either non-infected or TSWV-infected.

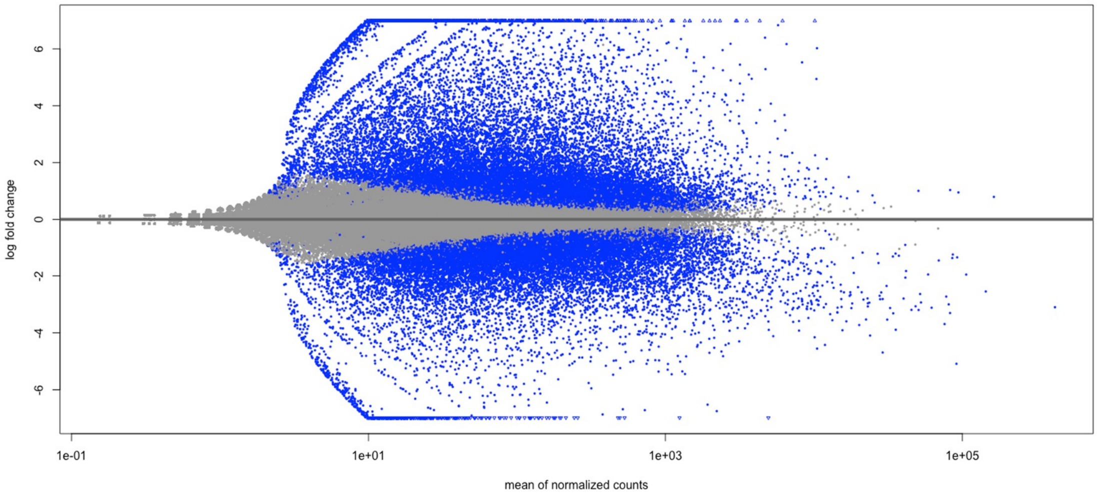

Fig. S6. MA plot for SunOleic 97R. SunOleic 97R MA-plot detailing the separation of differentially expressed genes with a p-value ≤ 0.001 and $\log_2 FC \geq 4$.

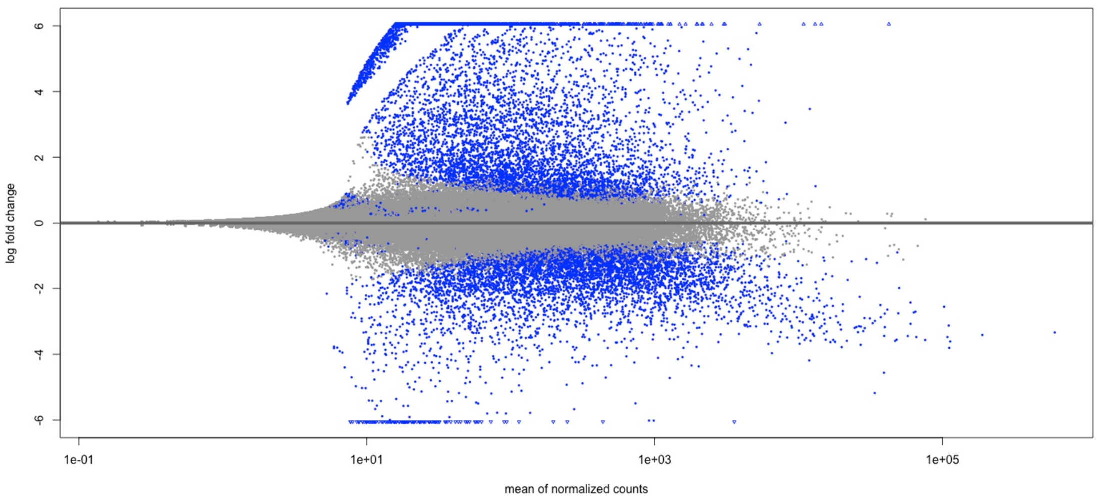

Fig. S7. MA plot for Tifguard. Tifguard MA-plot detailing the separation of differentially expressed genes with a p-value ≤ 0.001 and $\log_2$FC ≥ 4.

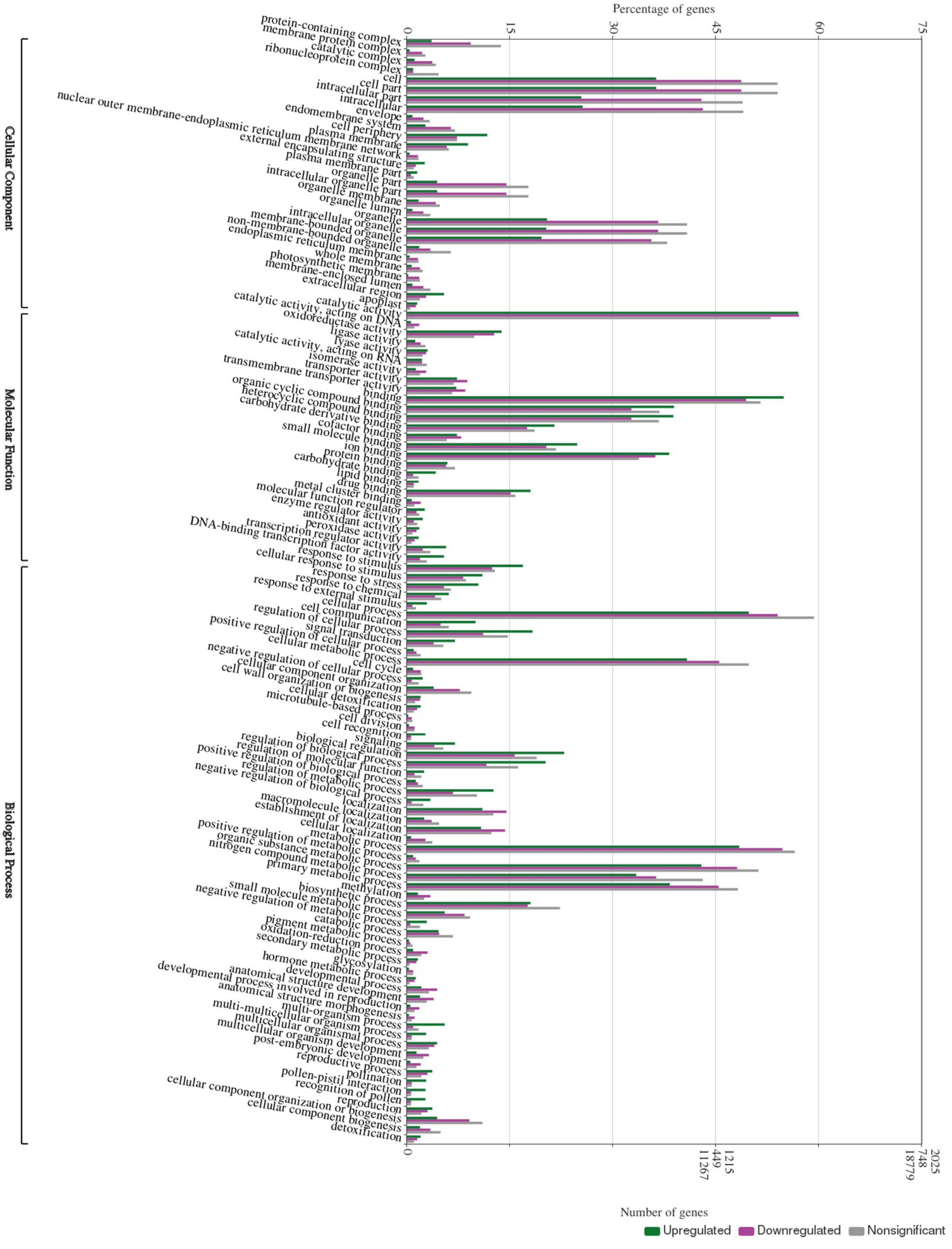

Fig. S8. SunOleic 97R GO terms. Gene Ontology (GO) terms assigned to differentially expressed genes (DEGs) that were upregulated and downregulated for the SunOleic 97R cultivar. Distribution of all significant level 2 & 3 GO terms present in this SunOleic 97R. Non-significant DEGs were included for comparison.

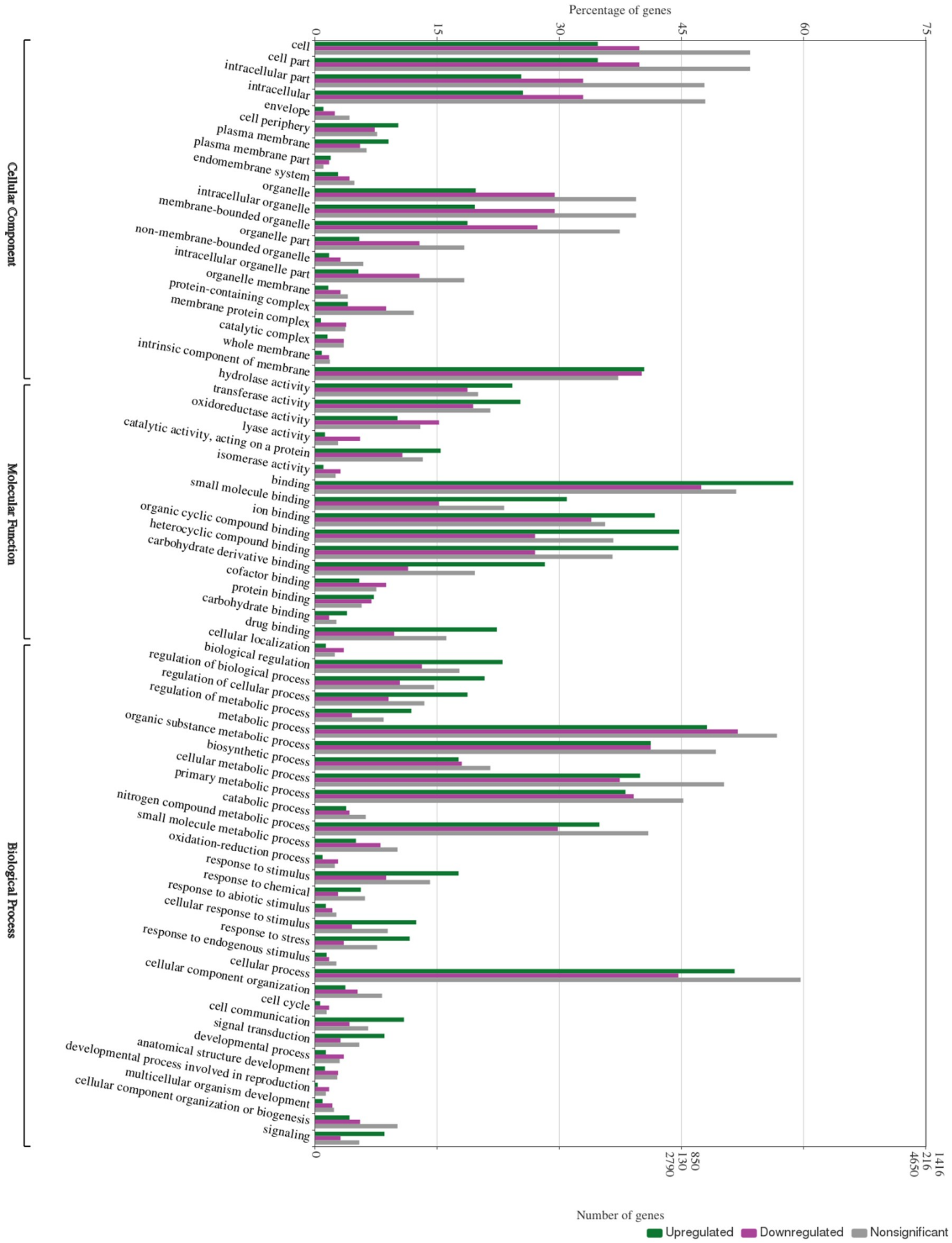

Fig. S9. Tifguard GO terms. Gene Ontology (GO) terms assigned to differentially expressed genes (DEGs) that were upregulated and downregulated for the Tifguard cultivar. Distribution of all significant level 2 & 3 GO terms present in this Tifguard. Non-significant DEGs were included for comparison.

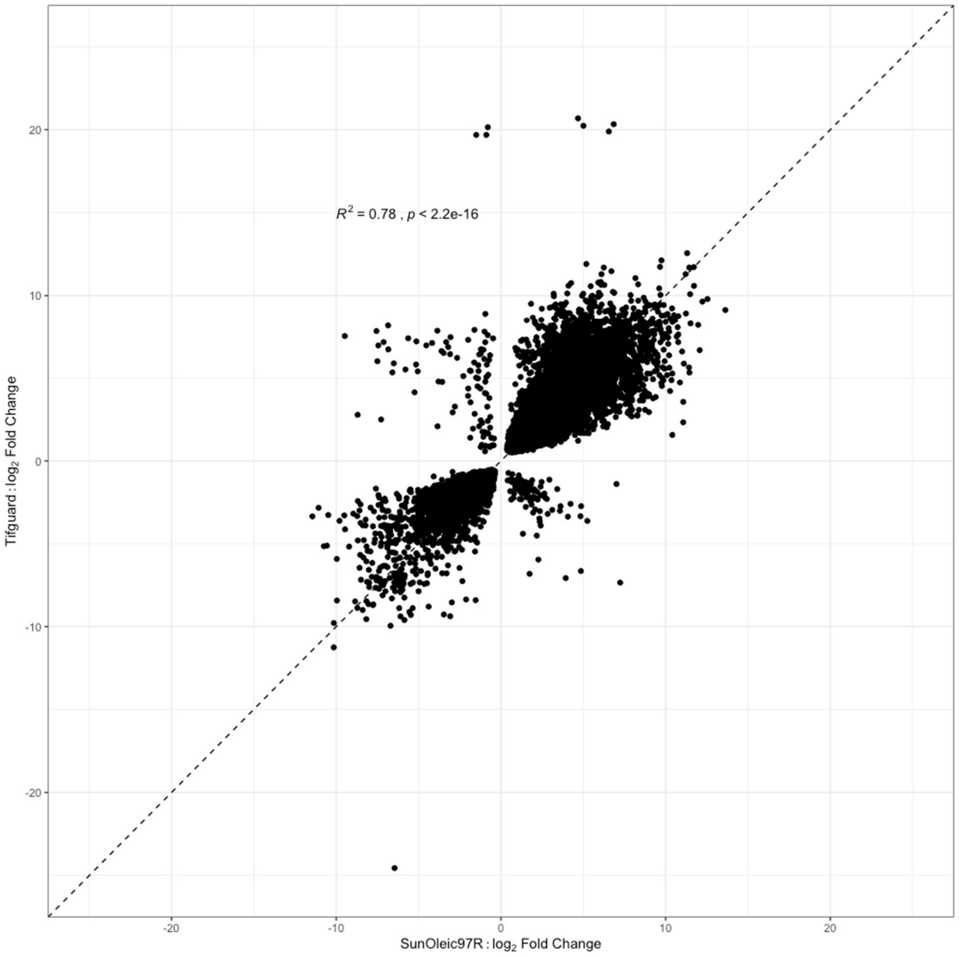

Fig. S10. Correlation of all DEGs between both cultivars. Pearson's correlation of all significant DEGs present in both cultivars. $R^2 = 0.78$ when considering all DEGs.

Table S1. DEG results from testing selective removal of samples. Total DEGs from selective removal of various samples. SunOleic 97R maintains stability after the selective removal of individual non-infected samples. Tifguard had a drop in total DEGs with the inclusion of a potentially confounded sample, which was removed for downstream analyses.

Cultivar	Total DEGs (> 4 log2FoldChange)
SunOleic	4,605
SunOleic (H_Sun_7 removed)	4,107
SunOleic (H_Sun_6 removed)	4,082
SunOleic (H_Sun_5 removed)	4,230
Tifguard	414
Tifguard (H_Tif_1 removed)	2,580

Table S2. SunOleic 97R Significantly Enriched GO Terms. Complete list of all significantly enriched GO terms from all GO categories (Levels 1 - 6) found in the SunOleic 97R cultivar.

GO ID	GO Terms	GO Term Description	Upregulation Gene Count	Downregulation Gene Count	Nonsignificant Gene Count	p-value
GO:0032991	Cellular Component	protein-containing complex	96	93	3423	<0.001
GO:0098796	Cellular Component	membrane protein complex	10	22	673	<0.001
GO:1902494	Cellular Component	catalytic complex	30	37	1047	<0.001
GO:1990904	Cellular Component	ribonucleoprotein complex	24	9	1163	<0.001
GO:0005623	Cellular Component	cell	979	486	13516	<0.001
GO:0044464	Cellular Component	cell part	979	486	13516	<0.001
GO:0044464	Cellular Component	cell part	979	486	13516	<0.001
GO:0044424	Cellular Component	intracellular part	687	428	12236	<0.001
GO:0005622	Cellular Component	intracellular	691	430	12266	<0.001
GO:0031975	Cellular Component	envelope	21	24	825	<0.001
GO:0012505	Cellular Component	endomembrane system	73	64	1762	<0.001
GO:0071944	Cellular Component	cell periphery	315	73	1826	<0.001
GO:0005886	Cellular Component	plasma membrane	239	58	1535	<0.001
GO:0042175	Cellular Component	nuclear outer membrane-endoplasmic reticulum membrane network	12	16	423	<0.001
GO:0030312	Cellular Component	external encapsulating structure	71	13	251	<0.001
GO:0044459	Cellular Component	plasma membrane part	41	6	259	0.023
GO:0044422	Cellular Component	organelle part	120	145	4440	<0.001
GO:0044446	Cellular Component	intracellular organelle part	118	145	4437	<0.001
GO:0031090	Cellular Component	organelle membrane	46	42	1204	<0.001
GO:0043233	Cellular Component	organelle lumen	22	24	854	<0.001
GO:0043226	Cellular Component	organelle	550	365	10228	<0.001
GO:0043229	Cellular Component	intracellular organelle	549	365	10219	<0.001
GO:0043227	Cellular Component	membrane-bounded organelle	530	355	9482	<0.001
GO:0044422	Cellular Component	organelle part	120	145	4440	<0.001
GO:0043228	Cellular Component	non-membrane-bounded organelle	49	34	1613	<0.001
GO:0098796	Cellular Component	membrane protein complex	10	22	673	<0.001
GO:0005789	Cellular Component	endoplasmic reticulum membrane	12	16	423	<0.001
GO:0044459	Cellular Component	plasma membrane part	41	6	259	0.023

GO:0031090	Cellular Component	organelle membrane	46	42	1204	<0.001
GO:0098805	Cellular Component	whole membrane	19	19	1204	<0.001
GO:0005886	Cellular Component	plasma membrane	239	58	1535	<0.001
GO:0042175	Cellular Component	nuclear outer membrane-endoplasmic reticulum membrane network	12	16	423	<0.001
GO:0005789	Cellular Component	endoplasmic reticulum membrane	12	16	423	<0.001
GO:0034357	Cellular Component	photosynthetic membrane	5	18	481	<0.001
GO:0031974	Cellular Component	membrane-enclosed lumen	22	24	854	<0.001
GO:0043233	Cellular Component	organelle lumen	22	24	854	<0.001
GO:0005576	Cellular Component	extracellular region	145	28	488	<0.001
GO:0048046	Cellular Component	apoplast	40	13	113	<0.001
GO:0003824	Molecular Function	catalytic activity	1539	570	13281	<0.001
GO:0140097	Molecular Function	catalytic activity, acting on DNA	16	18	275	0.004
GO:0016491	Molecular Function	oxidoreductase activity	373	127	2466	<0.001
GO:0016874	Molecular Function	ligase activity	33	20	688	<0.001
GO:0016829	Molecular Function	lyase activity	82	28	577	0.041
GO:0140098	Molecular Function	catalytic activity, acting on RNA	59	22	732	0.043
GO:0016853	Molecular Function	isomerase activity	34	28	481	0.005
GO:0005215	Molecular Function	transporter activity	198	88	1692	0.026
GO:0022857	Molecular Function	transmembrane transporter activity	194	85	1660	0.042
GO:0005488	Molecular Function	binding	1483	493	12886	0.001
GO:0097159	Molecular Function	organic cyclic compound binding	1051	326	9202	0.002
GO:1901363	Molecular Function	heterocyclic compound binding	1048	326	9191	0.002
GO:0097367	Molecular Function	carbohydrate derivative binding	581	175	4655	0.001
GO:0048037	Molecular Function	cofactor binding	197	79	1444	<0.001
GO:0036094	Molecular Function	small molecule binding	669	203	5439	0.001
GO:0043167	Molecular Function	ion binding	1032	361	8465	<0.001
GO:0005515	Molecular Function	protein binding	159	57	1748	0.037
GO:0030246	Molecular Function	carbohydrate binding	114	9	425	<0.001
GO:0008289	Molecular Function	lipid binding	45	10	262	0.013
GO:0008144	Molecular Function	drug binding	486	151	3956	0.009
GO:0051540	Molecular Function	metal cluster binding	19	20	277	0.003
GO:0098772	Molecular Function	molecular function regulator	71	14	443	0.004

GO:0030234	Molecular Function	enzyme regulator activity	63	10	385	0.002
GO:0016209	Molecular Function	antioxidant activity	48	14	212	<0.001
GO:0004601	Molecular Function	peroxidase activity	46	11	163	<0.001
GO:0140110	Molecular Function	transcription regulator activity	155	23	861	<0.001
GO:0003700	Molecular Function	DNA-binding transcription factor activity	147	19	725	<0.001
GO:0050896	Biological Process	response to stimulus	456	124	3193	<0.001
GO:0051716	Biological Process	cellular response to stimulus	296	82	2165	<0.001
GO:0006950	Biological Process	response to stress	281	54	1607	<0.001
GO:0042221	Biological Process	response to chemical	166	41	1258	0.015
GO:0009605	Biological Process	response to external stimulus	77	8	321	<0.001
GO:0009987	Biological Process	cellular process	1345	539	14842	<0.001
GO:0007154	Biological Process	cell communication	269	49	1527	<0.001
GO:0051716	Biological Process	cellular response to stimulus	296	82	2165	<0.001
GO:0050794	Biological Process	regulation of cellular process	495	111	3681	<0.001
GO:0007165	Biological Process	signal transduction	189	39	1318	<0.001
GO:0048522	Biological Process	positive regulation of cellular process	26	14	494	0.001
GO:0044237	Biological Process	cellular metabolic process	1102	454	12481	<0.001
GO:0007049	Biological Process	cell cycle	25	20	515	<0.001
GO:0048523	Biological Process	negative regulation of cellular process	61	7	424	0.004
GO:0016043	Biological Process	cellular component organization	105	77	2365	<0.001
GO:0071554	Biological Process	cell wall organization or biogenesis	54	19	294	<0.001
GO:1990748	Biological Process	cellular detoxification	53	15	244	<0.001
GO:0007017	Biological Process	microtubule-based process	5	7	201	0.002
GO:0051301	Biological Process	cell division	7	11	287	<0.001
GO:0008037	Biological Process	cell recognition	74	6	144	<0.001
GO:0023052	Biological Process	signaling	189	40	1320	<0.001
GO:0007165	Biological Process	signal transduction	189	39	1318	<0.001
GO:0065007	Biological Process	biological regulation	617	157	4721	<0.001
GO:0050789	Biological Process	regulation of biological process	546	116	4061	<0.001
GO:0065009	Biological Process	regulation of molecular function	67	11	522	0.034
GO:0050789	Biological Process	regulation of biological process	546	116	4061	<0.001
GO:0050794	Biological Process	regulation of cellular process	495	111	3681	<0.001

GO:0048518	Biological Process	positive regulation of biological process	36	16	575	0.002
GO:0019222	Biological Process	regulation of metabolic process	339	67	2562	<0.001
GO:0048519	Biological Process	negative regulation of biological process	92	7	606	<0.001
GO:0051179	Biological Process	localization	297	145	3160	0.008
GO:0033036	Biological Process	macromolecule localization	68	36	1170	<0.001
GO:0051234	Biological Process	establishment of localization	291	143	3069	0.009
GO:0051641	Biological Process	cellular localization	15	27	925	<0.001
GO:0008152	Biological Process	metabolic process	1306	546	14153	<0.001
GO:0019222	Biological Process	regulation of metabolic process	339	67	2562	<0.001
GO:0009893	Biological Process	positive regulation of metabolic process	23	13	462	<0.001
GO:0071704	Biological Process	organic substance metabolic process	1159	480	12827	<0.001
GO:0006807	Biological Process	nitrogen compound metabolic process	901	362	10786	<0.001
GO:0044238	Biological Process	primary metabolic process	1035	453	12079	<0.001
GO:0044237	Biological Process	cellular metabolic process	1102	454	12481	<0.001
GO:0032259	Biological Process	methylation	43	34	629	0.002
GO:0009058	Biological Process	biosynthetic process	486	176	5594	<0.001
GO:0044281	Biological Process	small molecule metabolic process	148	84	2309	<0.001
GO:0009892	Biological Process	negative regulation of metabolic process	77	5	473	<0.001
GO:0009056	Biological Process	catabolic process	123	47	1668	<0.001
GO:0042440	Biological Process	pigment metabolic process	9	5	207	0.013
GO:0055114	Biological Process	oxidation-reduction process	23	30	514	<0.001
GO:0019748	Biological Process	secondary metabolic process	44	14	112	<0.001
GO:0070085	Biological Process	glycosylation	9	9	215	0.014
GO:0042445	Biological Process	hormone metabolic process	34	11	108	<0.001
GO:0048518	Biological Process	positive regulation of biological process	36	16	575	0.002
GO:0048522	Biological Process	positive regulation of cellular process	26	14	494	0.001
GO:0009893	Biological Process	positive regulation of metabolic process	23	13	462	<0.001
GO:0032502	Biological Process	developmental process	57	44	800	0.001
GO:0048856	Biological Process	anatomical structure development	52	39	724	0.002
GO:0003006	Biological Process	developmental process involved in reproduction	14	18	276	0.001
GO:0009653	Biological Process	anatomical structure morphogenesis	8	11	180	0.012

GO:0051704	Biological Process	multi-organism process	149	9	428	<0.001
GO:0044706	Biological Process	multi-multicellular organism process	75	7	180	<0.001
GO:0032501	Biological Process	multicellular organismal process	119	40	796	0.002
GO:0007275	Biological Process	multicellular organism development	38	32	596	0.001
GO:0009791	Biological Process	post-embryonic development	14	20	355	<0.001
GO:0044706	Biological Process	multi-multicellular organism process	75	7	180	<0.001
GO:0022414	Biological Process	reproductive process	101	30	528	<0.001
GO:0003006	Biological Process	developmental process involved in reproduction	14	18	276	0.001
GO:0009856	Biological Process	pollination	75	7	176	<0.001
GO:0009875	Biological Process	pollen-pistil interaction	74	6	145	<0.001
GO:0048544	Biological Process	recognition of pollen	74	6	144	<0.001
GO:0000003	Biological Process	reproduction	101	30	532	<0.001
GO:0022414	Biological Process	reproductive process	101	30	528	<0.001
GO:0048519	Biological Process	negative regulation of biological process	92	7	606	<0.001
GO:0048523	Biological Process	negative regulation of cellular process	61	7	424	0.004
GO:0009892	Biological Process	negative regulation of metabolic process	77	5	473	<0.001
GO:0071840	Biological Process	cellular component organization or biogenesis	118	91	2747	<0.001
GO:0016043	Biological Process	cellular component organization	105	77	2365	<0.001
GO:0044085	Biological Process	cellular component biogenesis	50	34	1222	<0.001
GO:0098754	Biological Process	detoxification	53	15	249	<0.001
GO:1990748	Biological Process	cellular detoxification	53	15	244	<0.001

Table S3. Tifguard Significantly Enriched GO Terms. Complete list of all significantly enriched GO terms from all GO categories (Levels 1 - 6) found in the Tifguard cultivar.

GO ID	GO Terms	GO Term Description	Upregulation Gene Count	Downregulation Gene Count	Nonsignificant Gene Count	p-value
GO:0005623	Cellular Component	cell	655	115	3310	<0.001
GO:0044464	Cellular Component	cell part	655	115	3310	<0.001
GO:0044464	Cellular Component	cell part	655	115	3310	<0.001
GO:0044424	Cellular Component	intracellular part	478	95	2964	<0.001
GO:0005622	Cellular Component	intracellular	482	95	2969	<0.001
GO:0031975	Cellular Component	envelope	18	7	258	<0.001
GO:0071944	Cellular Component	cell periphery	192	21	471	0.001

GO:0005886	Cellular Component	plasma membrane	170	16	388	<0.001
GO:0044459	Cellular Component	plasma membrane part	36	5	63	0.007
GO:0012505	Cellular Component	endomembrane system	53	12	296	0.001
GO:0043226	Cellular Component	organelle	372	85	2444	<0.001
GO:0043229	Cellular Component	intracellular organelle	371	85	2443	<0.001
GO:0043227	Cellular Component	membrane-bounded organelle	354	79	2318	<0.001
GO:0044422	Cellular Component	organelle part	102	37	1134	<0.001
GO:0043228	Cellular Component	non-membrane-bounded organelle	32	9	363	<0.001
GO:0044422	Cellular Component	organelle part	102	37	1134	<0.001
GO:0044446	Cellular Component	intracellular organelle part	101	37	1134	<0.001
GO:0031090	Cellular Component	organelle membrane	31	9	247	<0.001
GO:0032991	Cellular Component	protein-containing complex	76	25	749	<0.001
GO:0098796	Cellular Component	membrane protein complex	13	11	231	<0.001
GO:1902494	Cellular Component	catalytic complex	28	10	217	<0.001
GO:0031090	Cellular Component	organelle membrane	31	9	247	<0.001
GO:0005886	Cellular Component	plasma membrane	170	16	388	<0.001
GO:0098805	Cellular Component	whole membrane	16	5	113	0.012
GO:0031224	Cellular Component	intrinsic component of membrane	763	116	2305	0.031
GO:0098796	Cellular Component	membrane protein complex	13	11	231	<0.001
GO:0044459	Cellular Component	plasma membrane part	36	5	63	0.007
GO:0016787	Molecular Function	hydrolase activity	457	54	1240	<0.001
GO:0016740	Molecular Function	transferase activity	476	56	1331	0.001
GO:0016491	Molecular Function	oxidoreductase activity	191	44	798	0.002
GO:0016829	Molecular Function	lyase activity	22	16	175	<0.001
GO:0140096	Molecular Function	catalytic activity, acting on a protein	290	31	820	0.021
GO:0016853	Molecular Function	isomerase activity	18	9	157	<0.001
GO:0005488	Molecular Function	binding	1108	137	3207	<0.001
GO:0036094	Molecular Function	small molecule binding	583	44	1437	<0.001
GO:0043167	Molecular Function	ion binding	788	98	2205	<0.001
GO:0097159	Molecular Function	organic cyclic compound binding	844	78	2269	<0.001
GO:1901363	Molecular Function	heterocyclic compound binding	843	78	2264	<0.001
GO:0097367	Molecular Function	carbohydrate derivative binding	532	33	1217	<0.001
GO:0048037	Molecular Function	cofactor binding	102	25	466	0.004

GO:0005515	Molecular Function	protein binding	136	20	352	0.043
GO:0030246	Molecular Function	carbohydrate binding	74	5	159	0.004
GO:0008144	Molecular Function	drug binding	422	28	999	<0.001
GO:0051641	Biological Process	cellular localization	25	10	148	0.007
GO:0065007	Biological Process	biological regulation	434	38	1098	<0.001
GO:0050789	Biological Process	regulation of biological process	393	30	908	<0.001
GO:0050789	Biological Process	regulation of biological process	393	30	908	<0.001
GO:0050794	Biological Process	regulation of cellular process	353	26	833	<0.001
GO:0019222	Biological Process	regulation of metabolic process	223	13	518	<0.001
GO:0008152	Biological Process	metabolic process	908	150	3515	<0.001
GO:0019222	Biological Process	regulation of metabolic process	223	13	518	<0.001
GO:0071704	Biological Process	organic substance metabolic process	779	119	3050	<0.001
GO:0009058	Biological Process	biosynthetic process	332	52	1334	0.001
GO:0044237	Biological Process	cellular metabolic process	754	108	3110	<0.001
GO:0044238	Biological Process	primary metabolic process	719	113	2805	<0.001
GO:0009056	Biological Process	catabolic process	72	12	384	<0.001
GO:0006807	Biological Process	nitrogen compound metabolic process	659	86	2536	<0.001
GO:0044281	Biological Process	small molecule metabolic process	94	23	625	<0.001
GO:0055114	Biological Process	oxidation-reduction process	17	8	149	<0.001
GO:0050896	Biological Process	response to stimulus	333	25	877	<0.001
GO:0042221	Biological Process	response to chemical	105	8	381	0.046
GO:0009628	Biological Process	response to abiotic stimulus	24	6	163	0.003
GO:0051716	Biological Process	cellular response to stimulus	234	13	553	<0.001
GO:0006950	Biological Process	response to stress	219	10	471	<0.001
GO:0009719	Biological Process	response to endogenous stimulus	27	5	160	0.011
GO:0009987	Biological Process	cellular process	972	129	3695	<0.001
GO:0050794	Biological Process	regulation of cellular process	353	26	833	<0.001
GO:0044237	Biological Process	cellular metabolic process	754	108	3110	<0.001
GO:0051716	Biological Process	cellular response to stimulus	234	13	553	<0.001
GO:0016043	Biological Process	cellular component organization	70	15	507	<0.001
GO:0007049	Biological Process	cell cycle	12	5	87	0.023

GO:0007154	Biological Process	cell communication	205	12	403	<0.001
GO:0007165	Biological Process	signal transduction	160	9	335	<0.001
GO:0032502	Biological Process	developmental process	25	10	184	<0.001
GO:0048856	Biological Process	anatomical structure development	22	8	169	<0.001
GO:0003006	Biological Process	developmental process involved in reproduction	5	5	82	<0.001
GO:0007275	Biological Process	multicellular organism development	17	6	140	0.001
GO:0003006	Biological Process	developmental process involved in reproduction	5	5	82	<0.001
GO:0071840	Biological Process	cellular component organization or biogenesis	80	16	625	<0.001
GO:0016043	Biological Process	cellular component organization	70	15	507	<0.001
GO:0023052	Biological Process	signaling	160	9	335	<0.001
GO:0007165	Biological Process	signal transduction	160	9	335	<0.001

Table S4. SunOleic 97R versus Tifguard top 7 significant Tifguard DEGs. Top 7 genes that may be of interest for further investigation into plant defenses. There was a large difference between the two cultivars in terms of log_2 fold change for these 7 genes, with low expression in SunOleic 97R ($|log_2FC| > 4$) and high expression in Tifguard ($|log_2FC| > 18$).

Sequence	Description	GO Term	SunOleic 97R (log2FoldChange)	Tifguard (log2FoldChange)	SunOleic 97R (p-value)	Tifguard (p-value)
ArahypEVm002624t15	alpha-dioxygenase 1	P:response to oxidative stress; P:cellular oxidant detoxification; F:peroxidase activity; F:heme binding; F:dioxygenase activity	-0.176301354	20.22017417	0.552249904	1.83465E-08
ArahypEVm016924t2	enoyl-CoA delta isomerase 2, peroxisomal	P:fatty acid beta-oxidation; F:dodecenoyl-CoA delta-isomerase activity; F:enoyl-CoA hydratase activity; C:peroxisome	0.277318009	20.18973321	0.345620788	2.03812E-08
ArahypEVm015273t7	probable mitochondrial import inner membrane translocase subunit TIM21	P:protein import into mitochondrial matrix; P:seedling development; C:TIM23 mitochondrial import inner membrane translocase complex; C:integral component of membrane	0.659037124	20.14649111	0.060173216	2.36014E-08
ArahypEVm004051t7	galacturonosyltransferase 8	P:G protein-coupled receptor signaling pathway; P:pectin biosynthetic process; P:cell wall organization; F:GTPase activity; F:guanyl nucleotide binding; F:G-protein beta/gamma-subunit complex binding; F:polygalacturonate 4-alpha-galacturonosyltransferase activity; C:Golgi	1.980144987	20.13297133	0.428404435	2.27606E-08

		membrane; C:integral component of membrane				
ArahypEVm009157t6	stilbene synthase 3-like	P:biosynthetic process; F:trihydroxystilbene synthase activity	0.683152356	19.94528616	0.727805465	4.42086E-08
ArahypEVm017947t2	tRNA-intron endonuclease	P:nucleic acid phosphodiester bond hydrolysis; F:endonuclease activity; C:integral component of membrane	-0.8874339	19.68439819	0.045381553	1.25511E-07
ArahypEVm001550t6	pre-mRNA-splicing factor ATP-dependent RNA helicase DEAH10 isoform X1	P:regulation of RNA splicing; P:positive regulation of transcription by RNA polymerase I; F:RNA binding; F:RNA helicase activity; F:ATP binding; C:spliceosomal complex; C:nucleolus	1.884393315	18.93382575	0.153236792	2.95854E-07
